# Highlighting the role of CD44 in cervical cancer progression: immunotherapy’s potential in inhibiting metastasis and chemoresistance

**DOI:** 10.1007/s12026-024-09493-6

**Published:** 2024-05-31

**Authors:** Cayleigh de Sousa, Carla Eksteen, Johann Riedemann, Anna-Mart Engelbrecht

**Affiliations:** 1 Department of Physiological Sciences, Faculty of Science, Stellenbosch University, Mike de Vries Building, C/o Merriman and Bosman Street, Stellenbosch, 7600 South Africa; 2 Cancercare, Cape Town, South Africa

**Keywords:** Cervical cancer, Immunology, Metastasis, CD44, EMT, Immune checkpoints

## Abstract

Cervical cancer affects thousands of women globally with recurring high-risk HPV infections being at the centre of cervical pathology. Oncological treatment strategies are continually challenged by both chemoresistance and metastasis within patients. Although both work hand-in-hand, targeting their individual mechanisms could prove highly beneficial for treatment outcomes. Such targets include the metastatic-promoting stem cell marker, CD44, which is abundant in cervical cancer cells and is common to both chemoresistance and metastatic mechanisms. Seeing that many existing advanced-stage cervical cancer treatment regimes, such as platinum-based chemotherapy regimens, remain limited and are rarely curative, alternative treatment options within the field of immunology are being considered. The use of immune checkpoint inhibition therapy, which targets immune checkpoints, CTLA-4 and PD-1/PD-L1, has shown promise as an alternate standard of care for patients suffering from advanced-stage cervical cancer. Therefore, this review aims to assess whether immune checkpoint inhibition can mitigate the pathological effects of CD44-induced EMT, metastasis, and chemoresistance in cervical cancer patients.

## Introduction

Cervical cancer remains a major health concern affecting women globally [1]. Common associated risk factors for cervical cancer include prolonged oral contraceptive use, smoking, numerous sexual partners, poor nutritional habits [2], and human immunodeficiency virus (HIV) [3]. However, human papillomavirus infection (HPV) continues to be the primary cause of malignancy, specifically infection with high-risk HPV subtypes, such as HPV16 and HPV18 [4]. Cervical oncogenesis is primarily driven by high-risk HPV oncogenes, *E5*, *E6*, and *E7* as these oncogenes evade the immune system whilst simultaneously disrupting physiological host genome cellular functioning mechanisms [5]. Cervical cancer treatment remains a challenge that often requires the combined use of various treatment modalities such as radiation, surgery, and chemotherapy [1]. Treatment is largely dependent on the International Federation of Gynecology and Obstetrics (FIGO) staging system (I–IV) [1] as well as the extent of the disease [4]. The standard of care (SOC) for low-risk/early-stage cancer patients typically include surgical resection. Locally advanced cervical disease is more challenging to treat and often requires concurrent chemoradiation therapeutic approaches [6]. Platinum-based chemotherapeutic drugs, such as cisplatin and taxanes such as paclitaxel, are most commonly used for cervical malignancy and have proven most effective [6]. Their administration in advanced-stage patients, both individually and in combination, has been shown to increase patients’ overall survival [7]. The overall patient prognostic potential largely decreases as the disease becomes recurrent/metastatic [4]. Furthermore, despite the development of targeted therapies, chemotherapy remains essential for treatment; however, a high failure rate is observed in metastatic disease due to chemoresistance, which may often be random and unpredictable. It is therefore essential to continue the search for biomarkers that will be able to predict treatment response to ensure a better survival rate for these patients.

Cell surface molecules, such as the cluster of differentiation 44 (CD44^1^), have been identified in abundance in tumour cells. An increased risk for treatment resistance and metastasis is highly prevalent in patients with high tumour expression of CD44 [8, 9]. Recognising the emerging role of immune modulation in cancer therapy, immune checkpoint inhibitors (ICIs^2^) targeting cytotoxic T-lymphocyte-associated antigen-4 (CTLA-4) and programmed death 1 (PD-1) [10] offer a new strategy to counteract CD44-mediated treatment resistance mechanisms to reinforce immune surveillance within cervical cancer patients. The aim of this review is, therefore, to evaluate whether the pathological effects of CD44-induced metastasis, epithelial-to-mesenchymal transition (EMT), and chemoresistance within cervical cancer patients can be blunted by immune checkpoint inhibition therapy.

## Cervical cancer and metastasis

Metastasis is currently the number one cause of cancer-associated mortality and morbidity [11]. Metastasis is a process that utilises the bloodstream or lymphatic system to induce the formation of secondary tumours at distant organ/tissue sites around the body [12]. This multi-step process creates significant therapeutic challenges [13] and is accountable for 90% of invasive cancer-associated deaths [14]. Moreover, metastasis forms the crux of both early and late tumourigenesis [15].

**Fig. 1:**
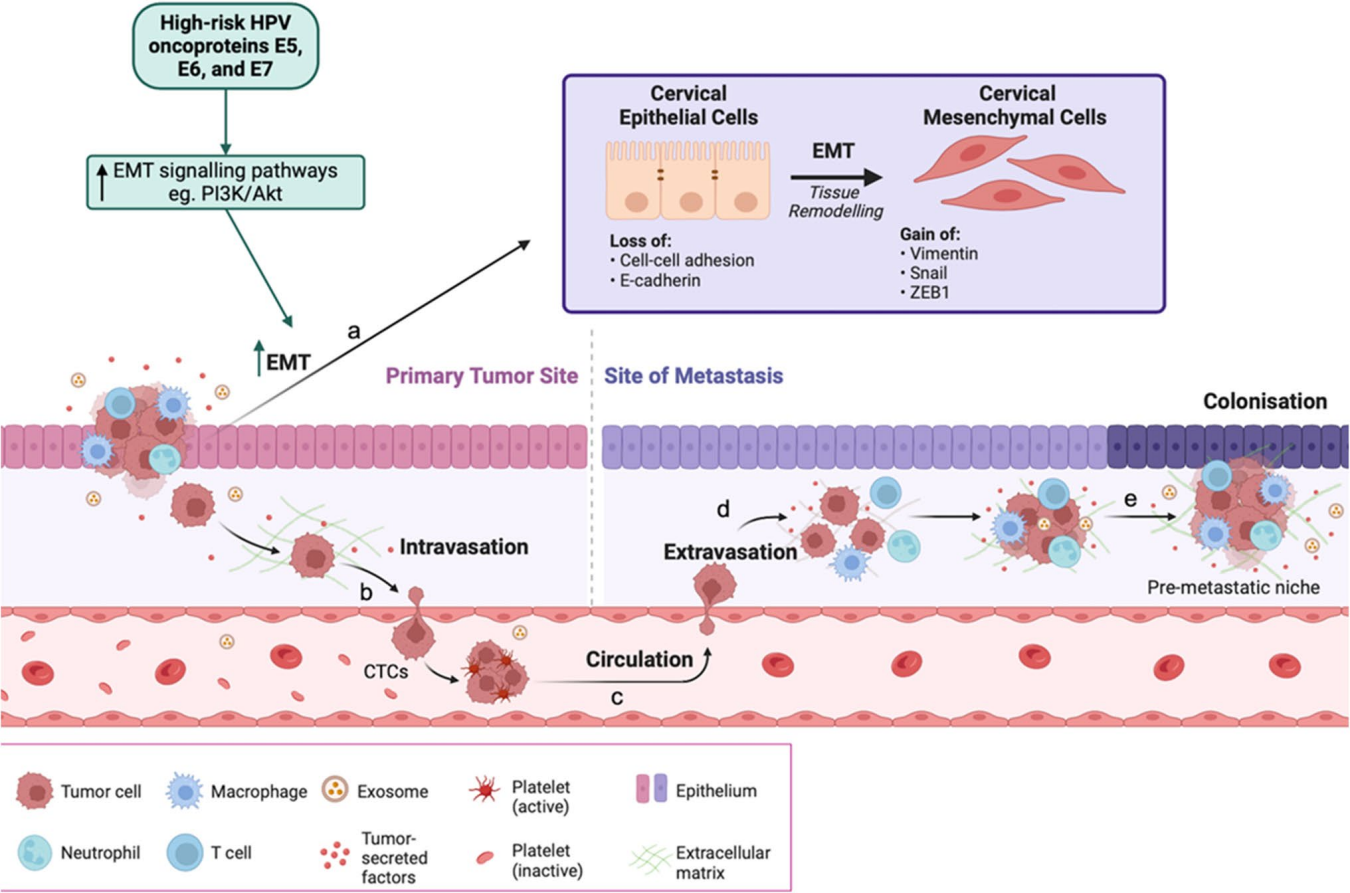
Representation of the metastatic cascade including the epithelial-to-mesenchymal transition (EMT) process (Biorender.com). (**a**) EMT of primary tumour cells, facilitated by microenvironment crosstalk mechanisms. The EMT process causes a decrease in epithelial markers, E-cadherin, Claudin-1, and ZO-1, and an increase in mesenchymal markers, vimentin, snail, N-cadherin, slug, beta-catenin, and ZEB1. EMT allows primary tumour cells to break away from their primary tumour site and migrate towards circulation. (**b**) Intravasation of primary tumour cells into circulation (blood/lymph), facilitated by ECM degradation. (**c**) In circulation, circulating tumour cells (CTCs) are directed towards a pre-metastatic niche/ target site. (**d**) Extravasation of tumour cells upon endothelial adherence and mesenchymal-to-epithelial transition (MET). Finally, (**e**) Colonisation and secondary tumour formation at a distant site from the primary tumour. Cervical cancer high-risk HPV oncoproteins E5, E6, and E7 play a role in enhancing the process of EMT via the upregulation of EMT-associated signalling pathway mechanisms at a), thereby further enhancing the metastatic cascade. Summary: The metastatic cascade is a complex process whereby cancer cells migrate from their original location to distant sites around the body. The cascade begins with an epithelial-to-mesenchymal cellular phenotypic transition (EMT), enabling cells to become more invasive to surrounding tissues. Subsequently, these cells enter the bloodstream (Intravasation) and travel to distant sites where they adhere to the walls of blood vessels (extravasation). Upon adherence, the cells revert to a more phenotypically stable state (MET) and eventually form secondary tumours. Certain viral proteins can expedite this process in cervical cancer by enhancing EMT. *Akt/PKB*, protein kinase B; *EMT*, epithelial-to-mesenchymal transition; *HPV*, human papilloma virus; *PI3K*, phosphatidylinositol 3-kinase; *ZEB1*, zinc-finger E-box-binding homeobox 1

The metastatic cascade is centralised around crosstalk mechanisms between tumour cells, stromal cells, immune cells, the extracellular matrix (ECM), and platelets [16]. In addition to crosstalk mechanisms, primary tumour cell heterogeneity should be considered [17] as these primary tumour sites are genetically and epigenetically unstable. This instability elevates the mutational frequency in tumour cells, resulting in treatment resistance and enabling them to metastasise [11]. The steps of metastasis are fivefold: (1) single cancer cell escaping from the primary tumour site via epithelial-to-mesenchymal transition (EMT) [15], (2) intravasation, (3) translocation into circulation as circulating tumour cells (CTCs), (4) extravasation, and finally (5) colonisation [16, 17]. However, not all metastatic tumour cells will successfully colonise at a secondary tumour site, only those primary tumour cells with metastatic potential, acquired through a succession of somatic mutations, will form secondary tumours [18] (summarised in Fig. 1).

It is therefore evident that the metastatic cascade is a central phenomenon in the progression of cervical cancer pathogenesis. With the facilitation of the EMT process and microenvironment crosstalk, the potential for treatment resistance within cervical cancer patients is potentially enhanced [13]. The cell surface molecule, CD44, is another protein highly involved in the EMT-induced metastatic process of cancer cells through its interaction with the tumour microenvironment (TME) [8]. Whilst CD44 is not mandatory for metastasis, it plays a critical role in contributing to metastasis [15] via its potential to enhance EMT-promoting pathway mechanisms. Namely, the transforming growth factor β (TGF-β), epidermal growth factor receptor (EGFR), phosphatidylinositol 3-kinase (PI3K), mitogen-activated protein kinase (MAPK), rat sarcoma (RAS), protein kinase B (Akt), nuclear factor kappa B (NF-kB), and wingless-related integration site (Wnt)/β-catenin signalling pathways [8]. It is the upregulation of such pathways that raises challenges when selecting an appropriate treatment for advanced/metastatic-stage cervical cancer patients.

## Cluster of differentiation 44 (CD44): cervical tumourigenesis stem cell marker

The multifunctional transmembrane cell surface adhesion receptor, CD44, promotes tumourigenesis via enhanced invasion and metastatic signalling pathways [19, 20]. This stem cell marker is ubiquitously expressed in tissues all around the body with CD44 protein abundance being tissue-specific [21]. Although CD44 is responsible for promoting various physiological functions such as cellular adhesion as well as communication between cells and the ECM [9], its aberrant overexpression gives rise to pathological occurrences such as tumour progression [22]. This pathology is mainly brought about by cancer-associated stem cells (CSCs) which are abundant in the TME and express high levels of CD44 on their surface. These CSCs, along with the structure of CD44, form the centre of CD44’s regulation and functional capacity [20].

###  CD44 structure

**Fig. 2:**
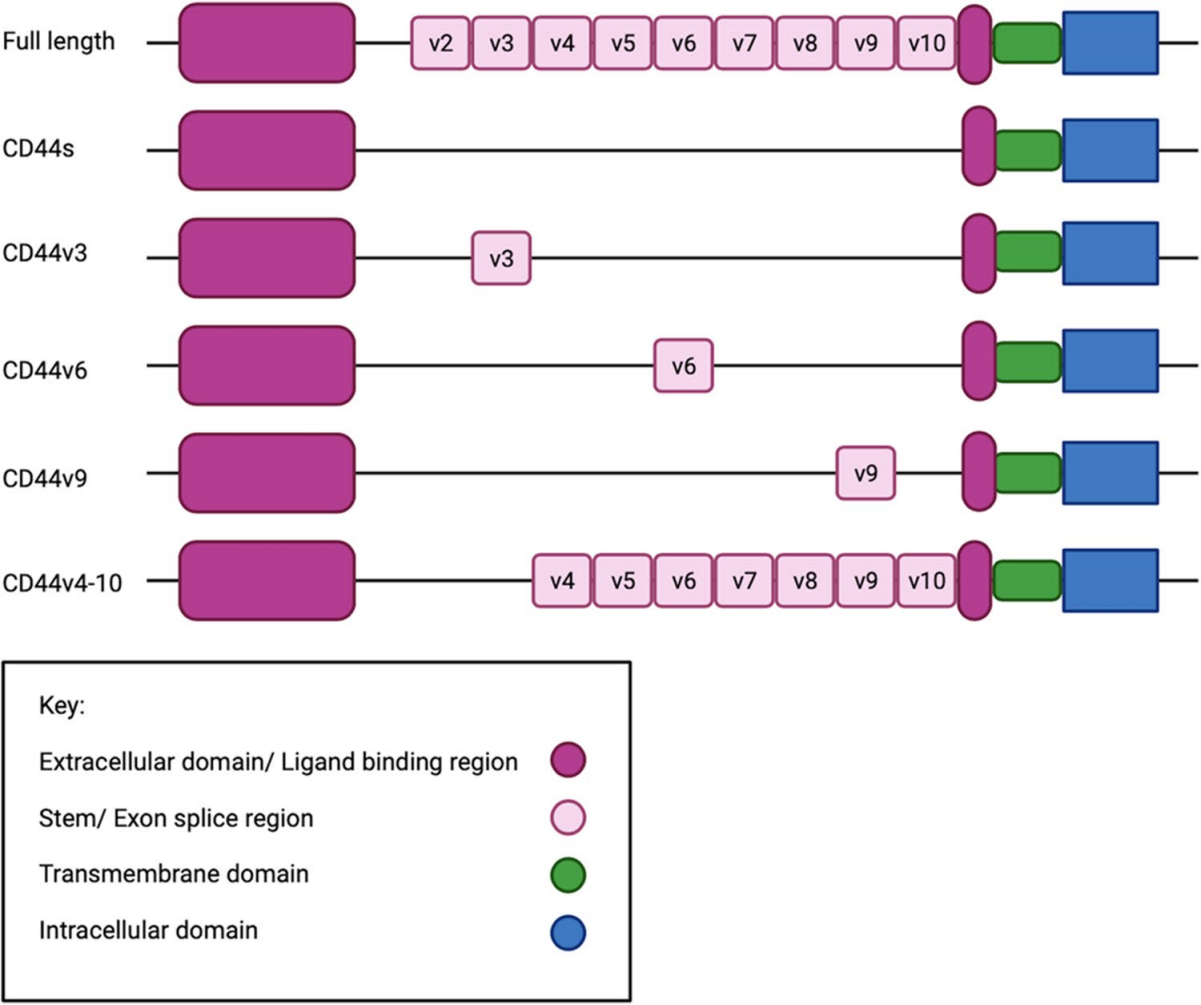
The structure of CD44s and its variant isoforms (not all) following exon splicing (Biorender.com). CD44, cluster of differentiation 44; CD44s, CD44 standard isoform; CD44v, CD44 variant isoform

The CD44 glycoprotein is composed of three structural domains [22], namely, the intracellular domain which is responsible for nucleic translocation and target gene transcription, the transmembrane domain, and the extracellular domain which is responsible for ligand binding and CD44 activation [20]. Although various structural isoforms of the CD44 protein exist. the two main isoforms include the CD44 standard (CD44s) and the CD44 variant (CD44v) isoform. However, ten different variant isoforms (v1–v10) can be expressed in various combinations within different types of cancers [9]. Even though these variants differ in function from that of CD44s, the basic structure of CD44s is kept in all variants where CD44s is the most abundantly expressed isoform [22]. Alternating RNA exon splicing within the extracellular domain’s stem region of CD44 [20] gives rise to variant functional and structural differentiation as illustrated in Fig. 2 [19].

The roles specific variants play in cancer pathogenesis are controversial and still need to be fully elucidated [8]. However, studies highlight the functions induced by different variants within specific types of cancers, specifically, the potential for the CD44v’s to enhance interactions with TME components via their additional binding motif availability [8]. In addition, some isoforms possess greater EMT and metastatic potential in comparison to others [8]. For example, CD44v6 is commonly observed in both colorectal and cervical cancer cells and is capable of enhancing EMT and invasion [23] (Table 1). Whereas, in breast cancer, the CD44s isoform is most commonly expressed and is therefore responsible for inducing metastasis within these patients [9].

**Table 1:** CD44 isoforms and their role in cervical cancer

CD44 isoform	Effect	Reference
CD44v6	Associated with high-risk HPV 16 E6 infection	[24]
CD44v6	Enhanced EMT and invasion	[19]
CD44v6	Lymph node metastasis and diagnostic indicator	[25]
CD44v6	Prognosticator of advanced early clinical-stage cervical cancer	[26]
CD44v7	High-risk HPV infection, invasion, and chemoresistance	[9] and [19] respectively
CD44v8		

In summary, CD44 comprises three structural domains responsible for enabling CD44 target gene transcription. Whilst the main isoforms include, CD44s and CD44v, ten variants (v1–v10) differing in both structure and function exist and can be generated from these isoforms. Various isoforms are observed in different types of cancers. For example, CD44v6 is commonly associated with cervical cancer and functions to enhance EMT-promoting mechanisms. Nonetheless, CD44 must first undergo ligand binding to initiate its metastatic and invasive signalling properties.

###  CD44 and metastasis

CD44’s communicative crosstalk between the ECM and the CSC surface induces metastatic signalling via the binding of various ligands, such as hyaluronic acid (HA^3^), osteopontin (OPN^4^), collagen, and matrix metalloproteinases (MMPs) [20]. Upon ligand binding, CD44 undergoes a conformational change through membrane type 1 matrix metalloprotease (MT1-MMP) and Presenilin-1/γ-secretase cleavage [8]. This cleavage allows for intracellular and extracellular domain release [21]. Once cleaved, the intracellular domain is free to translocate to the nucleus where it induces aberrant CD44 target gene transcription [8], including CD44 self-transcription [21]. Some of CD44’s target genes include *3-Phosphoinositide-dependent kinase 1* (*PDK1*^5^), *Fructose-bisphosphate aldolase* C (*ALDOC*^6^), *MMP-9*, and *6-phosphofructo-2-kinase/fructose-2,6-bisphosphatase 4* (*PFKFB4*^7^) [22], which are all involved in promoting cell survival, cell invasion, and metastatic cell signalling [21].

The CD44 binding ligand, HA, is highly abundant in the extracellular matrix ECM, favoured for HA-CD44 binding due to its prevalence in tumour cells and cancer-associated fibroblasts (CAFs) [22]. CD44 isoforms possess HA binding regions, amplifying HA-CD44 binding potential [8]. This interaction activates key signalling pathways including TGF-β, EGFR, PI3K, MAPK, RAS, Akt, NF-kB, and Wnt/β-catenin pathways, influencing cell adhesion, migration, survival, and proliferation [22]. CD44 also interacts with proteins like ezrin, radixin, and moesin (ERM^8^), modulating cell membrane protein uptake, angiogenesis (via increased vascular endothelial growth factor (VEGF)), and cell migration through MEK/extracellular signal-regulated kinase (ERK) signalling [27]. Furthermore, CD44 upregulates cyclin A and D, implicating its role in cell cycle regulation [22]. Taken together, these interactions promote EMT and CD44-induced metastasis resulting in enhanced treatment resistance capacity and poor prognosis in cancer patients [20].

## CD44 and treatment resistance

Despite advancements in cervical cancer management, metastasis and relapse remain significant challenges in advanced-stage cases [28]. Chemotherapeutic drugs can lead to the emergence of treatment-resistant cellular subclones, potentially through stress-induced phenotypical conversions involving somatic and genetic mutations, exacerbating EMT mechanisms [8]. The CSC surface receptor, CD44, is gaining recognition for its role in oncogenic processes, holding promise for prognostic and diagnostic applications [15].

### Role of CSCs in treatment resistance

CD44’s role in promoting treatment resistance in cervical cancer patients is linked to the self-renewal capacity of CSCs, which display high resistance to cytotoxic therapy [29, 30]. CSCs often remain static during chemotherapy, reducing drug efficacy and leading to tumour regeneration post-treatment [20]. Elevated CD44 levels are associated with poor prognosis and resistance to standard chemoradiation therapy [31]. Studies show that CSC markers, including CD44, correlate with increased resistance to radiation therapy and an increased EMT profile [31]. This resistance contributes to metastasis and patient relapse [28]. Furthermore, high-risk HPV oncogenes expressed by cervical cancer cells can also promote EMT, contributing to treatment resistance [28].

###  HPV, EMT, and treatment resistance

There is evidence of a direct link between treatment resistance in cervical disease and the involvement of HPV, specifically the overexpression of oncoproteins E6 and E7. HPV’s pathogenesis has a role to play as CD44-expressing cervical cancer stem cells (CCSCs^9^) have been identified as an ideal HPV target [28]. Upon viral entry, the proliferation of CCSCs via *E6* and *E7* oncogene expression occurs. In addition, both *E6* and *E7* possess EMT-promoting abilities [32]. Since CD44 expression is higher in cancer cells than in normal cells, this viral replicative process is therefore intensified [30]. Therefore, EMT-induced treatment resistance via high-risk HPV oncogene expression is prevalent in tumourigenesis.

As mentioned previously, HA ligand binding to CD44 can elicit various metastasis-promoting effects, which have also been observed in the promotion of treatment resistance in cancer patients.

###  CD44-HA treatment resistance

**Fig. 3:**
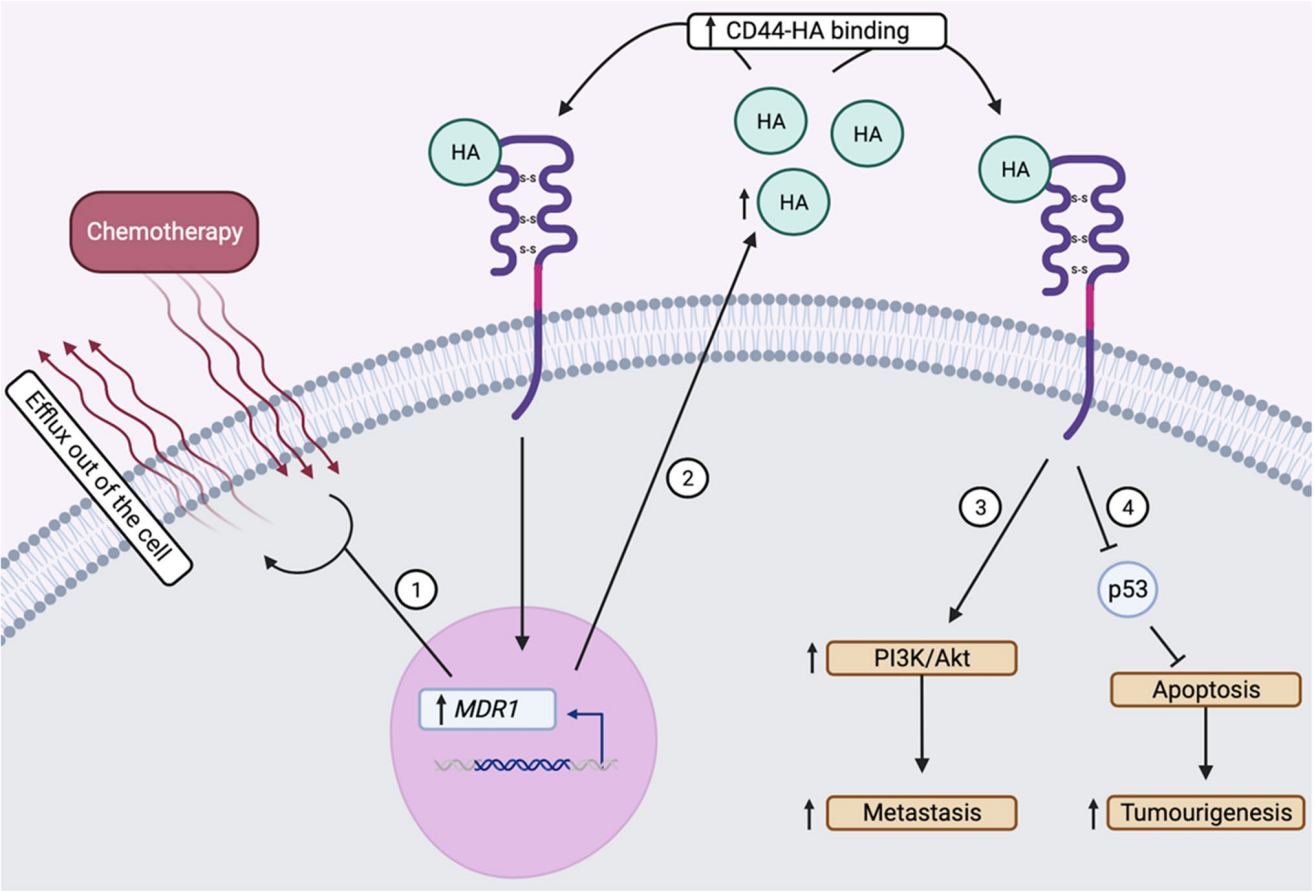
CD44-HA binding-induced treatment resistance mechanistic pathways (Biorender.com) Following HA binding to CD44, *MDR1* gene expression is upregulated. Enhanced *MDR1* levels (**a**) promote the efflux of chemotherapy drugs out of the cell thereby aiding in treatment resistance. Furthermore, increased *MDR1* expression (**b**) causes the upregulation of HA ligand concentration. The abundant HA ligand concentration enhances CD44-HA binding rates due to the high CD44 receptor expression levels. Moreover, CD44-HA binding will enhance treatment resistance metastatic pathways, (**c**) such as upregulated PI3k/Akt signalling. Lastly, CD44-HA binding causes the inhibition of tumour suppressor protein p53, (**d**) This results in decreased apoptosis and therefore enhances tumourigenesis. Summary: CD44-HA binding induces treatment resistance via enhanced chemotherapeutic efflux, increased CD44 self-binding potential, increased metastasis and EMT pathway signalling, and p53 apoptotic mechanism inhibition. Thus, fostering tumour progression and reinforcing treatment resistance within cancer cells. *Akt/PKB*, protein kinase B; *CD44*, cluster of differentiation 44; *HA*, hyaluronic acid; *PI3K*, phosphatidylinositol 3-kinase; *p53*, phosphorylated tumour suppressor protein 53; *MDR1*, multidrug resistance 1

An additional role player of treatment resistance occurs via CD44’s interaction with HA. CD44-HA binding facilitates chemoresistance through the upregulation of the *multidrug resistance 1* (*MDR1*^10^) gene, which encodes for P-glycoprotein [8]. Normally, *MDR1* ensures protection against cytotoxicity in both normal cells and CSCs. However, overexpression of CD44 amplifies *MDR1*’s effects and facilitates treatment resistance in over 50% of cancers [20]. This aberrant *MDR1* expression causes the efflux of chemotherapeutic drugs out of cancer cells and can reduce overall drug uptake [8]. For example, Paclitaxel-sensitive ovarian cancer cells showed lower levels of CD44 in comparison to those with high Paclitaxel resistance [20]. Moreover, *MDR1* enhances the production of HA, thereby promoting CD44-HA binding and consequently facilitating the activation of the metastatic PI3K/Akt signalling pathway. Therefore, it is evident that HA is at the core of CD44 pathogenesis and treatment resistance as illustrated in Fig. 3.

**Fig. 4:**
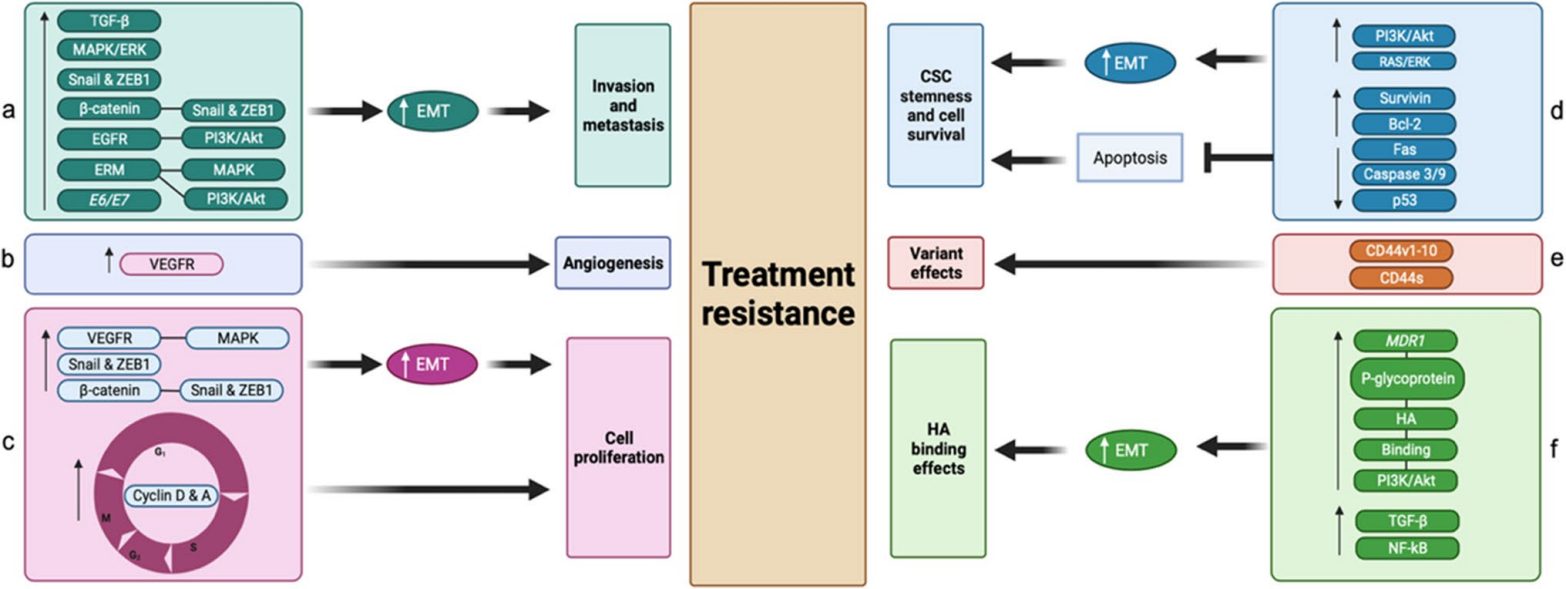
Representation of CD44’s main treatment resistance mechanisms (Biorender.com). The 6 main treatment resistance mechanisms induced by CD44 activity. (**a**) CD44 upregulates MAPK/ERK, snail, ZEB1, β-catenin, EGFR, ERM, PI3K/AKT, and *E6/E7* overexpression. These in turn upregulate EMT-induced invasion and metastasis. (**b**) CD44 upregulates VEGFR-induced angiogenesis. (**c**) CD44 upregulates VEGFR, snail, ZEB1, β-catenin, MAPK, and cell cycle regulators Cyclin D and A. This upregulation leads to EMT-induced cell proliferation as well as cell cycle-induced proliferation respectively. (**d**) CD44 upregulates PI3K/AKT, RAS/ERK, and anti-apoptotic Bcl-2, whilst downregulated apoptotic proteins Fas, Caspase 3/9, and tumour suppressor protein p53, thus, resulting in EMT-induced CSC stemness as well as cell survival via the inhibition of apoptosis. (**e**) CD44 variants have various effects in the induction of treatment resistance and can be cancer type specific. (**f**) CD44-HA binding promotes the upregulation of EMT-induced treatment resistance via *MDR1* gene expression, p-glycoprotein, HA (self-upregulation) binding, and PI3K/Akt signalling. *Akt/PKB*, protein kinase B; *Bcl-2*, B-cell lymphoma 2; *CD44*, cluster of differentiation 44; *CD44v*, variant isoform of cluster of differentiation 44; *CD44s*, standard isoform of cluster of differentiation 44; *CSC*, cancer stem cell; *EGFR*, epidermal growth factor receptor; *EMT*, epithelial-to-mesenchymal transition; *ERK*, extracellular signal-regulated kinase; *ERM*, ezrin, radixin, and moesin proteins; *HA*, hyaluronic acid; *MAPK*, mitogen-activated protein kinase; *MDR1*, multidrug resistance 1; *PI3K*, phosphatidylinositol 3-kinase; *P-glycoprotein*, phosphorylated glycoprotein; *p53*, phosphorylated tumour suppress or protein 53; *Ras*, rat sarcoma protein; *VEGFR*, vascular endothelial growth factor receptor; *ZEB1*, zinc-finger E-box-binding homeobox 1

Similarly, CD44-HA binding can lead to apoptotic inhibition mechanisms via its negative regulation of tumour suppressor protein p53, Fas (Fas cell surface death ligand), and caspase 3/9 [9]. Therefore, the role of various CD44 treatment resistance mechanisms involved in promoting tumourigenesis is abundant (Fig. 4).

In summary, the role CD44 plays in cervical cancer treatment resistance mechanisms originates from its association with high-risk HPV oncogenes and CSCs as these two factors promote EMT, self-renewal capacity, and reduced drug efficacy. Furthermore, CD44’s interaction with HA further exacerbates treatment resistance by increasing *MDR1* gene expression thus enhancing EMT-promoting pathways, such as PI3K/akt, and facilitating drug efflux. This complex interplay highlights the pivotal role CD44 plays in driving treatment resistance and tumour progression in cervical cancer.

###  CD44-targeted therapies

Despite attempts to treat advanced-stage cervical cancer patients with concurrent chemotherapy regimens, challenges with relapse and resistance persist, necessitating the exploration of alternative treatment options that target CD44’s metastatic mechanisms. Currently, the most frequently used CD44-targeted therapies to overcome treatment resistance include CD44-targeted vaccines, nanoparticle-mediated siRNA CD44 delivery, and anti-CD44 monoclonal antibodies [33]. Although many studies surrounding the potential for these therapies in the treatment of cancer exist, clinical trials focusing on the targeting of CD44 in cervical cancer patients are limited. However, clinical trials conducted on CD44v6-associated cancers, such as head and neck squamous cell carcinomas, are evident and could potentially serve as a foundation for future cervical cancer clinical trial studies (Table 2).

**Table 2:** Summary of clinical trials using CD44-targeted therapies to overcome treatment resistance in CD44v6-positive cancers

Clinical trial	Phase	Cancer type	Reference
CD44v6-Targeting Immunoconjugate Bivatuzumab	I	CD44v6-positive squamous cell carcinoma	[34]
Dose Escalation Study with Anti-CD44v6 Bivatuzumab	I	CD44v6-positive squamous cell carcinoma	[35]
186Re-Labeled Chimeric Monoclonal Antibody U36	I	CD44v6-positive squamous cell carcinoma	[36]
Safety and Pharmacokinetics of Bivatuzumab Mertansine	I	CD44v6-positive metastatic breast cancer	[37]

Therefore, it is observed that current CD44v6 clinical trials are investigating the potential benefits of the use of immunotherapeutic antibodies as an alternative treatment approach.

## Immunotherapy

Recurrent/metastatic cervical cancer remains a significant global health concern. Platinum-based chemotherapy, the primary treatment option, is limited and rarely curative [38]. Immune evasion has emerged as a novel hallmark of cancer since 2011, adding to the existing characteristics outlined by Weinberg and Hanahan in 2000 [39]. Tumour cells’ ability to manipulate the immune system presents ongoing therapeutic challenges.

**Fig. 5:**
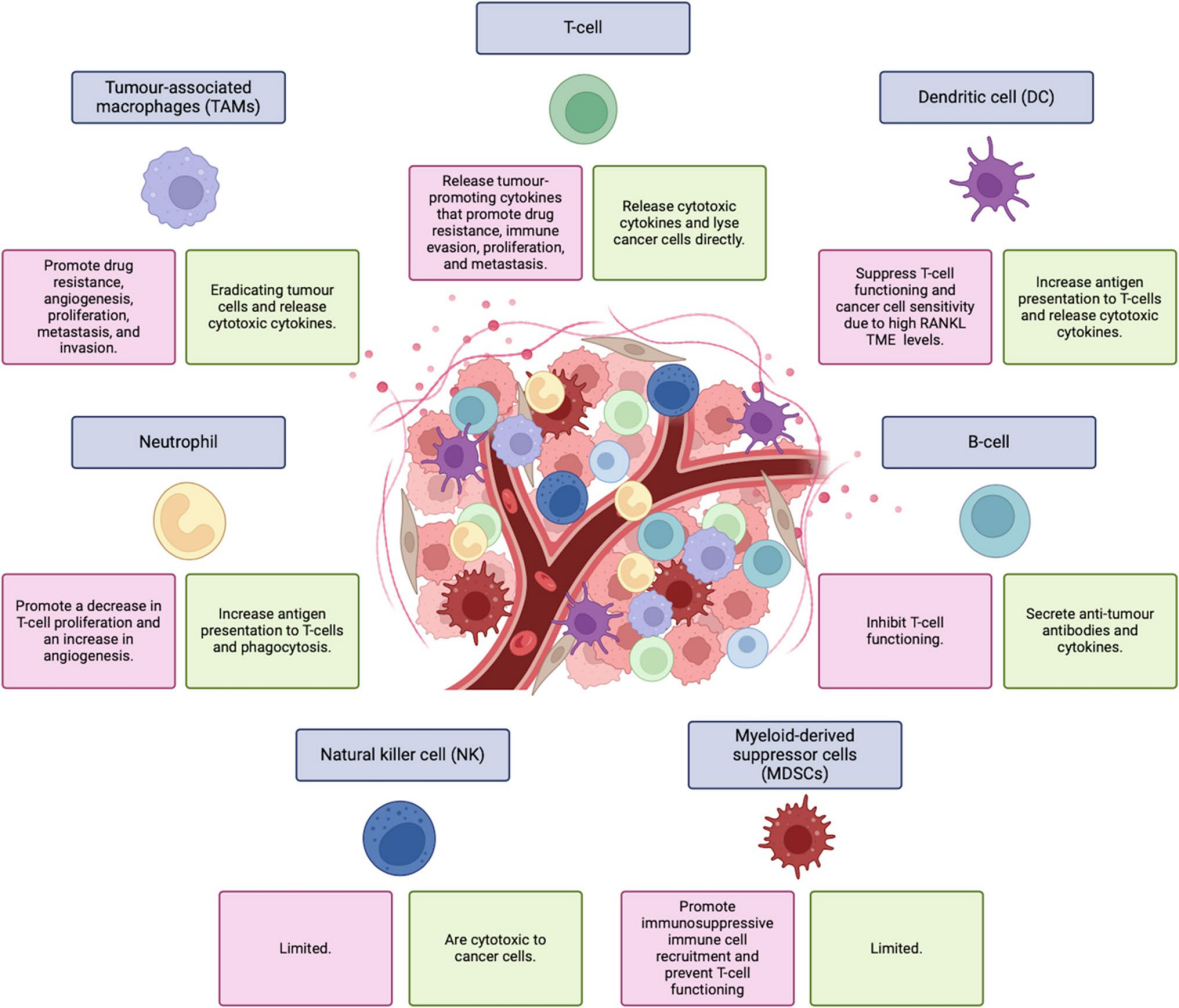
Representation of pro- and anti-tumour functioning of immune cells that are commonly found within the tumour microenvironment [45–48] (Biorender. com) where the pink blocks are representative of pro-tumour activities and the green blocks represent the anti-tumour activities of the immune cells. *RANKL*, receptor activator of nuclear factor kappa-Β ligand; *T-cell*, T-lymphocyte; *TME*, tumour microenvironment

In addition, strong communicative networks within the TME aid in treatment resistance as infiltrating immune cells promote tumour cell invasion and metastasis [40]. The TME is composed of a variety of different cell types, namely, CAFs, immune cells, cancer cells, stromal cells, and inflammatory cells. The production of chemokines, inflammatory markers, and cytokines from these TME cells functions to induce tumourigenesis through multiple crosstalk mechanisms [41] such as enhanced metastatic and EMT pathways [42]. Immune cells that are commonly found within the TME, and that promote immune infiltration, include natural killer (NK) cells, T-lymphocytes (T-cells), bone marrow-derived suppressor cells (MDSCs^11^), macrophages, neutrophils, tumour infiltrating lymphocytes (TILS), B-cells, and dendritic cells (DCs) [43]. However, not all immune cells promote immune surveillance (anti-tumour) but are responsible for inducing immune evasion/ignorance (pro-tumour) [44] as illustrated in Fig. 5.

Investigations into the use of immune system activating agents such as therapeutic vaccines, engineered and infiltrating T-cells, ICIs, and antibody-drug conjugates, are being brought to oncological attention [49]. Elevated overall survival rates [50] in both post-platinum chemotherapy and front-line treatments are highly required. Therefore, immunotherapy, through the introduction of ICIs, has revolutionised treatment for cervical disease [50] and will be the focus of this review.

###  Targeted blockade: immune checkpoint inhibitors

Immunotherapy is combating cancer by leveraging the innate potential of the immune system to identify and eradicate tumour cells directly [38]. Three main immune checkpoints of interest include programmed death 1 (PD-1), programmed cell death ligand 1 (PD-L1), and cytotoxic T-lymphocyte-associated antigen-4 (CTLA-4) which are largely involved as therapeutic targets [51]. Physiologically, both PD-1 and CTLA-4 inhibit T-cell activation in the immune system as a protective strategy for normal and healthy cells and against autoimmunity. However, both display upregulated expression patterns in neoplastic cells, thus enabling immune system hijacking and evasion [51]. Thus, the introduction of monoclonal antibodies in the form of ICIs to induce a ‘targeted blockade’ into treatment plans for metastatic disease patients, could prove to be highly effective against progressive metastatic disease. Moreover, several clinical trials investigating the use of ICIs in combating cervical cancer exist. Therefore, Table 3 presents a concise overview of selected cervical cancer clinical trials utilising common ICIs.

**Table 3:** Summary of common ICIs used in cervical cancer clinical trials

Clinical trial	Immune checkpoint inhibitor	Phase	Target	Outcomes	Limitations	Reference
KEYNOTE-028 (Phase II currently ongoing)	Pembrolizumab	Ib	PD-L1	• 17% overall response rate • ~ 9-month overall survival rate	Adverse side effects	[52]
CheckMate 358	Nivolumab	I/II	PD-1	• 26.3% objective response rate • ~ 21.9-month overall survival rate	Treatment related adverse effects: 63.2% of patients	[53]
GOG 9929	Ipilimumab	I	CTLA-4	• 74% presented with a 1-year disease-free survival rate	Adverse side effects	[54]
MEDI4736 (Currently ongoing)	Durvalumab Tremelimumab	I	PD-L1 CTLA-4	• 6 out of the 13 cervical cancer patients currently present with stable disease	Adverse side effects	[55]

Furthermore, another important immune attenuating phenomenon to consider is T-cell exhaustion. T-cell exhaustion occurs within the TME in response to the upregulation of PD-1 and CTLA-4 expression. Interestingly, a study performed by Heeren et al. (2019) observed a more ‘recoverable’ state of exhaustion within T-cells of the cervical cancer tumour microenvironment, following PD-1 blockade [56]. Thus, highlighting the potential of ICIs to combat advanced-stage disease through T-cell revitalisation [57]. However, not all patients benefit from ICI therapy due to their specific cancer makeup [58]. Thus, an additional factor to consider when selecting an appropriate treatment is the role CD44 plays in promoting EMT and the metastatic cascade. Evidence supporting their individual and combined relationship with ICI therapy is scarce and needs to be further investigated.

###  Stem cell marker: CD44, and the role of immune checkpoint inhibitors

The stem cell marker, CD44, is responsible for instigating a communicative network between CSCs and the TME [59]. This communication often prompts treatment resistance, as CD44 is highly associated with metastatic and EMT-inducing pathways [60]. Moreover, CD44 offers an additional role within the immune system, that has the potential to aid in immune escape and to promote tumour progression [60, 61]. Thus, the potential of immunotherapy to blunt the combined treatment resistance effects of CD44 could prove highly beneficial.

CSCs are known for their self-renewal potential which often results in increased treatment resistance within patients [30]. Furthermore, CSCs can function as immunosuppressive drivers within the TME via crosstalk mechanisms with tumour-associated macrophages (TAMs), T-cells, and MDSCs. For example, CD44-positive CSCs display an inverse relationship with CD8 + T-cell expression levels thereby favouring T-cell-induced tumour-killing inhibition [62]. In addition, evidence supporting the ability of CSCs to bypass ICI therapy exists as CSCs possess the potential to express immune checkpoints, CTLA-4, and PD-L1 on their surface [63]. For instance, CD44-positive CSCs have evolved adaptive immune resistance by engaging the CTLA-4 receptor on T-cells [63]. Although little evidence supports the direct role CSCs and ICIs have on cervical cancer specifically, potential conclusions can be drawn based on the known presence of high CD44 levels within cervical tumours.

Due to the known increased CD44 expression in cancer cells [30], a study conducted on bladder cancer by Liu et al. (2023) showed that patients presenting with high CD44 levels, have a poor prognosis [59]. They also encouraged the possibility of CD44 acting as a positive regulator of PD-L1, as PD-L1 expression levels were seen to be correlated to CD44 expression. Zhang et al. (2020), found similar findings amongst several cervical cancer cell lines, where cells expressing high CD44 levels, expressed simultaneously also high PD-L1 levels [61]. Moreover, patients who had been treated with chemotherapy prior to this study, displayed elevated PD-L1 expression levels. Thus, suggesting the potential role high levels of PD-L1 have on cervical cancer progression [61].

Furthermore, Liu et al. (2023) speculated from their study results that CD44 expression may affect immune checkpoint CTLA-4 expression levels [59]. Their speculations were confirmed through the findings of Gutiérrez-Hoya et al. (2019) where CTLA-4 levels were seen to be upregulated in various immortal cervical cancer cell lines, namely, HeLa, CaSki, and C33A (HPV-negative cell line) [60]. Interestingly, they observed both the CaSki and HeLa cervical cancer cell lines with corresponding high levels of CD44 in conjunction with the high CTLA-4 levels. However, CD44 levels were not observed in the C33A cell line thus proposing that the virus may possess control over CD44 expression. Therefore, evidence supporting the involvement of CD44 in promoting enhanced immune evasion potential through the upregulated expression of both PD-L1 and CTLA-4 in cervical cancer cells is evident. Further investigations into the role of ICI therapy on CD44’s EMT-inducing potential within cervical cancer, could show promising therapeutic potential.

###  Quartet of cervical cancer treatment resistance

**Fig. 6:**
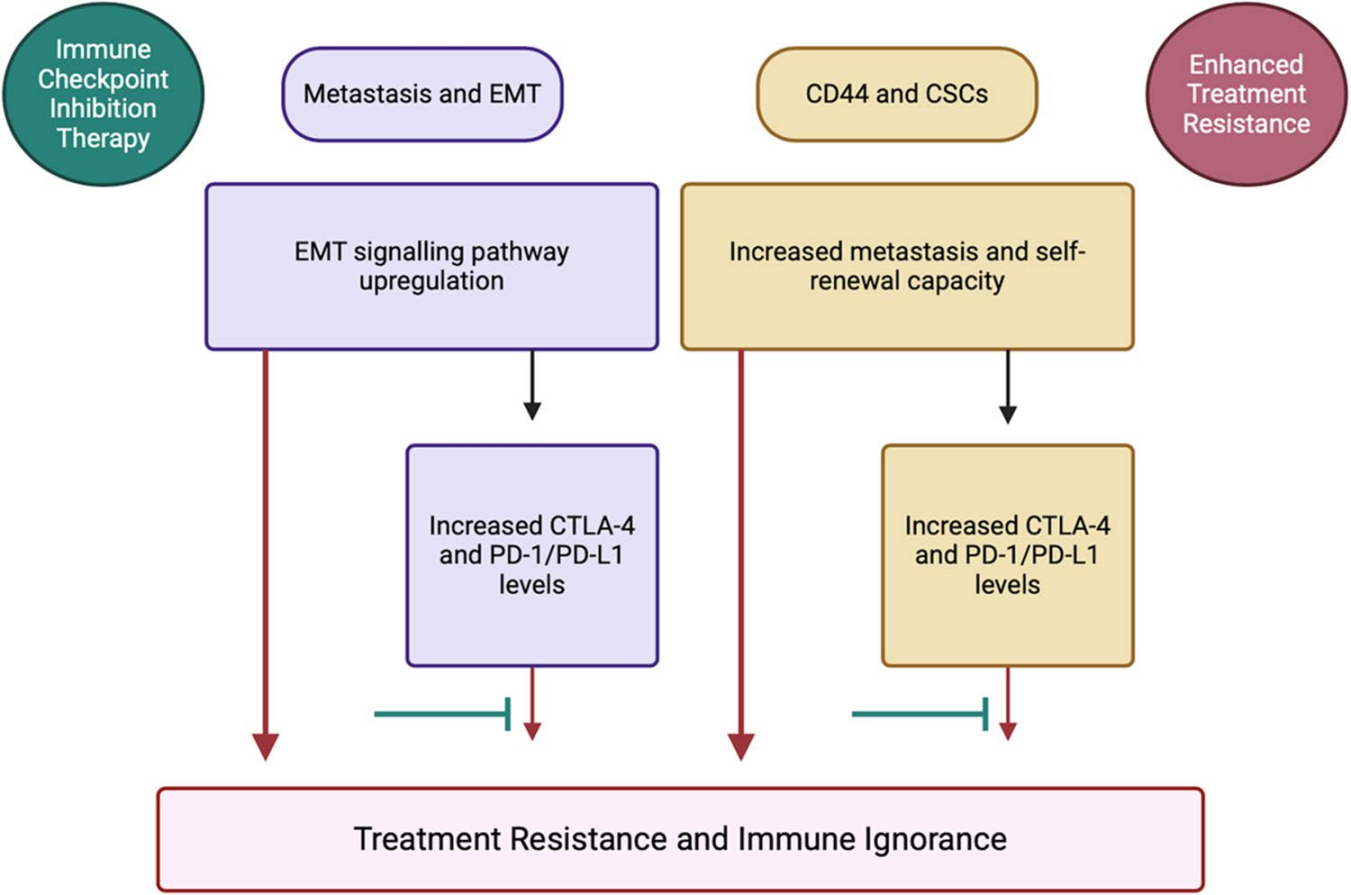
A diagrammatic summary of the cervical cancer treatment resistance mechanisms and the inhibiting effect ICIs exerts on these processes (Biorender.com) Increased levels of EMT signalling pathway molecules (e.g. TGF-β) cause upregulation of immune checkpoints. Therefore, treatment with ICIs could prove effective. CD44 and CSCs go hand-in-hand when promoting immune ignorance and treatment resistance as they enhance metastatic pathways. However, evidence supporting their potential to upregulate immune checkpoint expression in cancer cells is evident, thereby allowing for the potential treatment with ICIs. Summary: Cervical cancer’s treatment resistance mechanisms entail unregulated EMT signalling and increased immune checkpoint expression levels. This creates potential for the effective use of ICIs. Moreover, CD44 and CSCs further contribute to immune evasion and resistance but may also upregulate immune checkpoints, thus creating an opportunity for ICI treatment. *CD44*, cluster of differentiation 44; *CSCs*, cancer-associated stem cells; *CTLA-4*, cytotoxic T-lymphocyte-associated antigen-4; *EMT*, epithelial-to-mesenchymal transition; *PD-1*, programmed death 1; *PD-L1/2*, programmed death ligand

Thus far, evidence supporting the treatment resistance potential of metastasis, EMT, CD44, and CSCs, have been discussed in this review (Fig. 6). Furthermore, the intertwining roles and effects each treatment resistance inducer exerts on one another to promote cervical tumourigenesis were also brought to light. Investigations into the probable use of immunotherapy as a viable treatment option in combating this resistance through the use of ICIs, CTLA-4 and PD-1/PD-L1, were sought out as an adjuvant to the typical concurrent chemotherapy standard of care for patients suffering from advanced-stage cervical cancer.

## Conclusion

Further research is needed to understand the role of metastasis-inducing EMT in advanced-stage cervical cancer, particularly in conjunction with CD44 and immune checkpoints. The interplay between EMT and immune cells in cancer is complex and influenced by factors such as the TME and individual immune responses. Ongoing studies aim to unravel these complexities and develop more personalised and targeted therapies to modulate the immune response and inhibit EMT-induced metastasis.

## Data Availability

No datasets were generated or analysed during the current study.

## Footnotes

1 **CD44 –** Cluster of differentiation 44.

2 **ICIs** – Immune checkpoint inhibitors.

3 **HA –** Hyaluronic acid.

4 **OPN -** Osteopontin.

5 ***PDK1–3****-Phosphoinositide-dependent kinase*.

6 ***ALDOC*****-***Fructose-bisphosphate aldolase C*.

7 ***PFKFB4–6****-Phosphofructo-2-kinase/fructose-2,6-bisphosphatase 4*.

8 **ERM -** Ezrin, radixin, and moesin.

9 **CCSC –** Cervical cancer stem cell.

10 ***MDR1*****–***Multidrug resistance 1 gene*.

11 **MDSC -** Bone marrow-derived suppressor cells.
